# Constructing Layered/Tunnel Biphasic Structure via Trace W-Substitution in Tunnel-Type Cathode for Elevating Sodium Ion Storage

**DOI:** 10.3390/molecules30102175

**Published:** 2025-05-15

**Authors:** Wenjing Shi, Hengxiang Li, Zihan Wang, Lingyang Liu, Yixin Feng, Rui Qiao, Ding Zhang, Haibo Li, Zhaoyang Wang, Pengfang Zhang

**Affiliations:** 1 Shandong Provincial Key Laboratory of Chemical Energy Storage and Novel Cell Technology, School of Chemistry and Chemical Engineering, Liaocheng University, Liaocheng 252059, China; 13156168783@163.com (Z.W.); liulingy0425@163.com (L.L.); 14768274256@163.com (Y.F.); 15143911968@163.com (R.Q.); haiboli@mail.ustc.edu.cn (H.L.); wzy9218@126.com (Z.W.); zhangpengfang111@163.com (P.Z.); 2 School of Chemical Engineering and Pharmacy, Wuhan Institute of Technology, Wuhan 430205, China; zhangding@wit.edu.cn

**Keywords:** oxide cathode, layered/tunnel biphasic structure, tungsten substitution, phase transition, sodium-ion batteries

## Abstract

Tunnel-type Na_0.44_MnO_2_ is extensively regarded as an appealing cathode for sodium-ion batteries due to its cost-effectiveness and excellent cycling performance. However, low theoretical capacity, resulting from insufficient Na^+^ storage sites, hinders its practical application. Herein, the strategy of constructing a tunnel-phase-dominated layered/tunnel biphasic compound was proposed via trace W-substitution and the co-precipitation method. Experimental analysis reveals that W-introduction can effectively redistribute electronic configuration, induce tunnel-to-layered structure evolution, accelerate Na^+^ (de)intercalation kinetics, and enhance structural stability. The optimized layered/tunnel Na_0.44_Mn_0.99_W_0.01_O_2_ cathode integrates the superiorities of the layered and tunnel structures, delivering a high capacity of 153.1 mAh g^−1^ at 0.1 C and outstanding cycle life, with 71% capacity retention over 600 cycles at 5 C. Significantly, the full cell assembled with the Na_0.44_Mn_0.99_W_0.01_O_2_ cathode and a commercial hard carbon anode exhibits a competitive energy density of 183.2 Wh kg^−1^, along with a remarkable capacity retention of 75.5% over 200 cycles at 1 C. This work not only highlights the superior sodium storage performance of biphasic composites owing to the synergistic effects between layered and tunnel structures, but also unveils new possibilities for constructing high-performance hybrid cathodes that predominantly consist of the tunnel phase using a suitable design strategy.

## 1. Introduction

Sodium-ion batteries (SIBs) have been considered as potential electrochemical energy storage systems owing to the abundance of sodium resources, as well as their environmental benignity, high safety, and similar operating mechanism to that of lithium-ion batteries (LIBs) [1,2,3,4,5]. The electrochemical performance of SIBs, i.e., specific capacity, rate performance, and cycle life, is largely determined by the structure of the cathode materials. To date, numerous cathode materials have been explored, including transition metal oxides, polyanionic compounds, and Prussian blue analogs [6,7,8]. Among these, tunnel-type Na_0.44_MnO_2_ (NMO) and layered Na_x_TMO_2_ (NTMO, TM = transition metal, x ≤ 1) are recognized as two of the most promising cathode candidates on account of their cost-effectiveness, simple synthesis process, and decent sodium storage performance [9,10]. The tunnel-structured NMO features a unique S-shaped three-dimensional channel framework, composed of edge-sharing MnO_6_ octahedra and corner-sharing MnO_5_ square pyramids (Figure S1a). There are three sodium sites, in which Na1 occupies small hexagon tunnels, and Na2 and Na3 are located in big S-shaped tunnels. The Mn ions within the structure are situated in two environments: all the Mn^4+^ and half of the Mn^3+^ occupy a MnO_6_ octahedral area, while the remaining Mn^3+^ present in the MnO_5_ square pyramidal shape [11,12]. This arrangement can effectively mitigate lattice strain, alleviate volume fluctuations, and accelerate Na^+^ diffusion kinetics, contributing to impressive cycling stability and rate performance. Additionally, tunnel-type NMO exhibits excellent air stability and can operate in aqueous electrolytes due to its resistance to forming hydrated phases [13]. Nevertheless, the reversible capacity of NMO cathode is always restricted by the inherent phase transition between the sodium-poor Na_0.22_MnO_2_ and sodium-rich Na_0.66_MnO_2_ phases, leading to a low theoretical capacity of approximately 121 mAh g^−1^, hindering its practical application in SIBs [14].

The layered NTMO cathodes are primarily categorized into P2- and O3-type structures, depending on the sodium coordination environment and the stacking sequence of the oxygen layer. Among them, the “P” and “O” refer to the position of Na in the trigonal prismatic and octahedral sites, respectively, while the “2” and “3” stand for the number of oxygen layers stacked in a unit cell [15,16]. Compared to O3-type cathodes, P2-type materials typically offer superior cycling stability and rate performance, benefiting from their structural integrity and direct Na^+^ diffusion paths (Figure S1b). In contrast, O3-type cathodes tend to exhibit higher capacity due to their sufficient Na content [17,18]. Nonetheless, the layered NTMO cathodes usually undergo complicated phase evolution during the Na^+^ extraction/insertion processes, such as P2↔O2 for the P2 phase or O3→O3′→P3→P3′→P3″ for the O3 phase, resulting in large volume variations, structural degradation, and severe capacity fade throughout prolonged cycling [19,20]. Additionally, layered NTMO cathodes are sensitive to moisture, with H_2_O and CO_2_ molecules penetrating the lattice structure, causing an unstable structure and poor cycling performance [21,22].

To tackle these challenges, various methods have been explored to enhance the sodium storage performance of tunnel-type NMO and layered NTMO cathodes. For instance, element substitution (e.g., Mg [23,24], Fe [25,26], Li [27,28], Nb [29,30], Ti [31,32], Mo [33,34], F [35,36], etc.) has been widely employed to improve structural stability, inhibit complex phase transitions, and facilitate reaction kinetics. Recently, biphasic composites have gained considerable attention, providing a promising approach to designing high-performance cathode materials. Studies have shown that biphasic structures, such as P2/tunnel [37,38], P2/O3 [39,40], and P2/P3 [41,42] composites, can combine the structural advantages of individual phases, mitigate their inherent defects, and result in a significant enhancement in the overall electrochemical performance through synergistic effects between different phase structures. However, it remains a great challenge to design a tunnel-phase-dominated layered/tunnel compound cathode with low Na content, high capacity, admirable cycle life, and rate performance. Furthermore, a deeper comprehension of the formation process, structural evolution, and synergistic mechanisms is needed for further exploration.

In this study, the hybrid materials with layered/tunnel structures are successfully constructed using trace tungsten (W) ion substitution through a co-precipitation method. The influences of the W-doping ratio, calcination temperature, and time on the growth process, crystal structure, micro morphology, phase evolution, and Na^+^ storage mechanism of NMO were systematically investigated, combining experimental characterization with density functional theory (DFT) analysis. As a result, when the W-doping content reaches 1%, the Na_0.44_Mn_0.99_W_0.01_O_2_ (NMOW1) cathode with an optimal structure ratio (93.78% tunnel phase) displays a high discharge capacity (153.1 mAh g^−1^, 0.1 C), remarkable rate performance, and superior cycling stability (93.6 mAh g^−1^ and 71% capacity retention after 600 cycles at 5 C). DFT analysis reveals that the W-introduction effectively improves the metallicity of the material and accelerates electron transfer. The reversible structural transition of NMOW1 is further verified by ex situ X-ray diffraction (XRD) during the Na^+^ insertion/extraction process. Additionally, the assembled NMOW1//HC full cell demonstrates an impressive energy density of 183.2 Wh kg^−1^. This study provides valuable insights for constructing tunnel-structure-predominated composite materials with high capacity and excellent cycle life through a rational synthesis strategy.

## 2. Results and Discussion

**Scheme 1:**
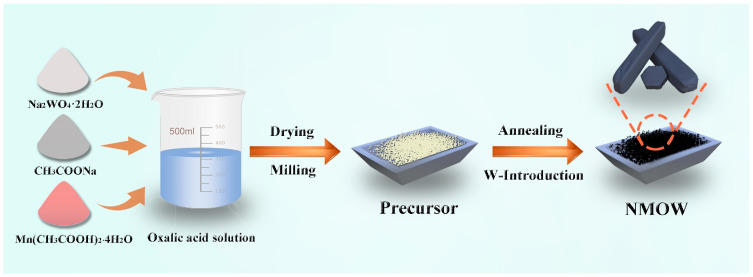
The synthetic diagram of the NMOW samples.

W-substituted Na_0.44_Mn_1−x_W_x_O_2_ (x = 0.005, 0.01, 0.015, and 0.02, called NMOW0.5, NMOW1, NMOW1.5, and NMOW2, respectively) cathodes were prepared via the co-precipitation method, and the schematic diagram is shown in Scheme 1.

**Figure 1:**
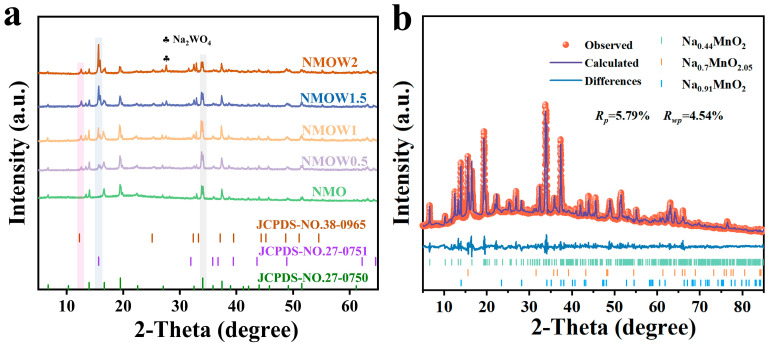
(**a**) XRD patterns of NMO, NMOW0.5, NMOW1, NMOW1.5, and NMOW2. (**b**) Rietveld refinement patterns of XRD for NMOW1.

The structural characterization of the NMO and NMOW samples was investigated using XRD. The diffraction peaks of NMO correspond to the typical tunnel structure (*Pbam*, JCPDS No. 27-0750) (Figure 1a). For the NMOW samples, the XRD patterns indicate a hybrid structure that includes tunnel structure (*Pbam*, JCPDS No. 27-0750), a P2-type layered structure (*P6_3_/mmc*, JCPDS No. 27-0751), and small amounts of Na_0.91_MnO_2_ (JCPDS No. 38-0965) [43,44]. As the W content increases from 0.5% to 2% (Figure 1a), the intensity of the (002) diffraction peak for the P2-type layered structure increases significantly, while the diffraction peak for the tunnel structure remains detectable, indicating that the hybrid structure is maintained. In addition, a noticeable diffraction peak corresponding to the impurity phase Na_2_WO_4_ (*Fd-3m*, JCPDS No. 12–0772) appears when the W-doping content reaches 1.5% and 2%. The Rietveld refinement results and the lattice parameters for the NMOW1 cathode are shown in Figure 1b and Table S1. Compared to those of NMO (*a* = 9.0810, *b* = 26.4513, and *c* = 2.8229 Å), the lattice parameters *a* and *c* in the NMOW1 cathode increase slightly to 9.0812 and 2.8232 Å, and the *b* value slightly decreases to 26.4482 Å. This result suggests that the W^6+^ enters the transition metal layer and exhibits a strong repulsion from Na^+^, leading to a slightly expanded layer distance [14,45]. According to the Rietveld refinement results, the composition of NMOW1 is calculated to be 93.78% NMO (tunnel-type), 4.82% Na_0.7_MnO_2.05_ (P2-type), and 1.40% Na_0.91_MnO_2_, indicating that the tunnel structure predominates in the NMOW1 sample. This finding is significantly different from the results reported by Ding et al., where a 1% W-doping ratio led to a complete phase transformation from the tunnel to the layered structure [14]. This discrepancy likely stems from the differences in preparation methods, yielding distinct structural configurations. Given the low content and poor performance of Na_0.91_MnO_2_ in SIBs, its contribution to the electrochemical performance is not considered in subsequent analyses. Additionally, the structural evolution under different annealing times (3, 6, 9, 12, and 15 h) and temperatures (700, 800, 900, and 1000 °C) for NMOW1 was investigated. The result demonstrates that the W-doped samples maintain the hybrid structure throughout the synthesis process. The characteristic peaks of both the tunnel and P2 phase can be found from 3 to 15 h at 900 °C, and the primary phase, namely the tunnel phase, displays a good crystallinity at 6 h (Figure S1c). The intensity of the (002) peak for the P2 phase is notably enhanced when the temperature reaches 1000 °C (Figure S1d), signifying good crystallinity for the layered structure under this condition, while the (350) plane and other peaks for the tunnel phase are more pronounced at 900 °C. This phenomenon indicates that the calcination temperature exerts an important influence on the resulting phase components. Therefore, the optimal preparation conditions were determined to be 900 °C for 6 h.

**Figure 2:**
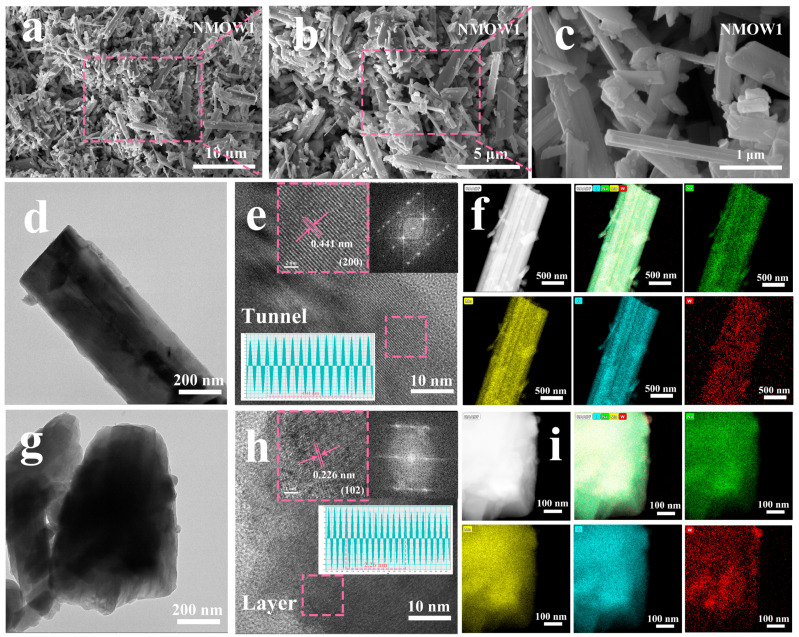
(**a**–**c**) SEM images. (**d**–**i**) TEM, high-resolution TEM images, and EDS mappings for NMOW1 cathode, with rod-like and plate-like structures.

The morphologies of the NMOW1 material were examined by scanning electron microscopy (SEM) at various magnifications. The images show a biphasic hybrid structure composed of rod-like particles and a small number of plate-like particles, further demonstrating that the tunnel structure is mainly dominant in the layered/tunnel hybrid structure (Figure 2a–c). In contrast, the NMO material consists solely of rod-like particles with a mean width and length of approximately 2 and 4 μm (Figure S2). When the W-doping content increases from 0.5% to 2%, the morphology of the NMOW samples still retains the biphasic structure. However, the NMOW2 exhibits irregularly shaped polygonal plate-like particles with less distinct rod-like particles (Figure S2). Further analysis of the NMOW1 material at different annealing times and temperatures (Figure S3) reveals a similar tunnel and layered hybrid structure. Notably, polygonal plate-like particles become more prominent when the calcination temperature reaches 1000 °C, correlating with an increase in the layered phase content, which is consistent with the XRD results. The crystal structure of the NMOW1 sample was further analyzed by transmission electron microscopy (TEM) and high-resolution TEM. The *d*-spacing of 0.441 nm in Figure 2e is attributed to the (200) plane of the tunnel phase, while the interplanar spacing of 0.226 nm in Figure 2h corresponds to the (102) plane of the P2 layered structure. The well-defined lattice fringes demonstrate the high crystallinity of the NMOW1 sample. Energy dispersive spectroscopy (EDS) mapping manifests a homogeneous distribution of all elements across both rod-like and plate-like particles (Figure 2f,i), confirming W-incorporation into the layered/tunnel hybrid structure.

**Figure 3:**
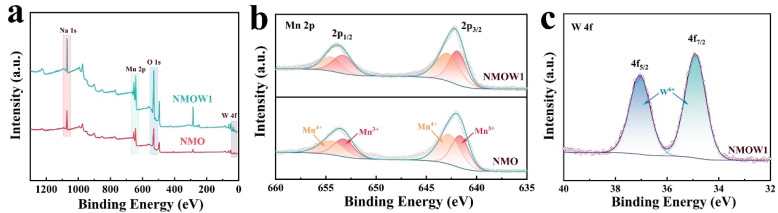
(**a**) XPS survey spectra of NMOW1 and NMO samples. High-resolution XPS of Mn 2p (**b**) and W 4f (**c**).

To investigate the surface composition, X-ray photoelectron spectroscopy (XPS) spectra were obtained for the NMOW1 and NMO samples. The XPS survey spectra exhibit three characteristic peaks at 1071, 642, and 530 eV, representing the presence of Na, Mn, and O elements in both the NMO and NMOW1 cathodes. The peak at 35 eV in NMOW1 belongs to the W-element (Figure 3a). The Mn 2p high-resolution XPS spectra show two broad peaks at approximately 642.2 and 654.1 eV, assigned to Mn 2p_3/2_ and Mn 2p_1/2_, respectively [43,46,47] (Figure 3b). Moreover, each peak can be further deconvoluted into two distinct peaks associated with Mn^3+^ (641.7 and 653.3 eV) and Mn^4+^ (642.8 and 654.4 eV), suggesting the coexistence of Mn^3+^ and Mn^4+^ in both NMO and NMOW1 [14,47,48]. Particularly, the semi-quantitative ratio of Mn^3+^/Mn^4+^ increases from 0.81 in NMO to 0.87 in NMOW1 owing to the charge compensation. As illustrated in Figure 3c, the W 4f XPS spectra display two prominent peaks at 35 and 37 eV, which can be categorized as W 4f_7/2_ and W 4f_5/2_, respectively [14,49], demonstrating the successful introduction of the W^6+^ ion into the NMOW1 sample.

**Figure 4:**
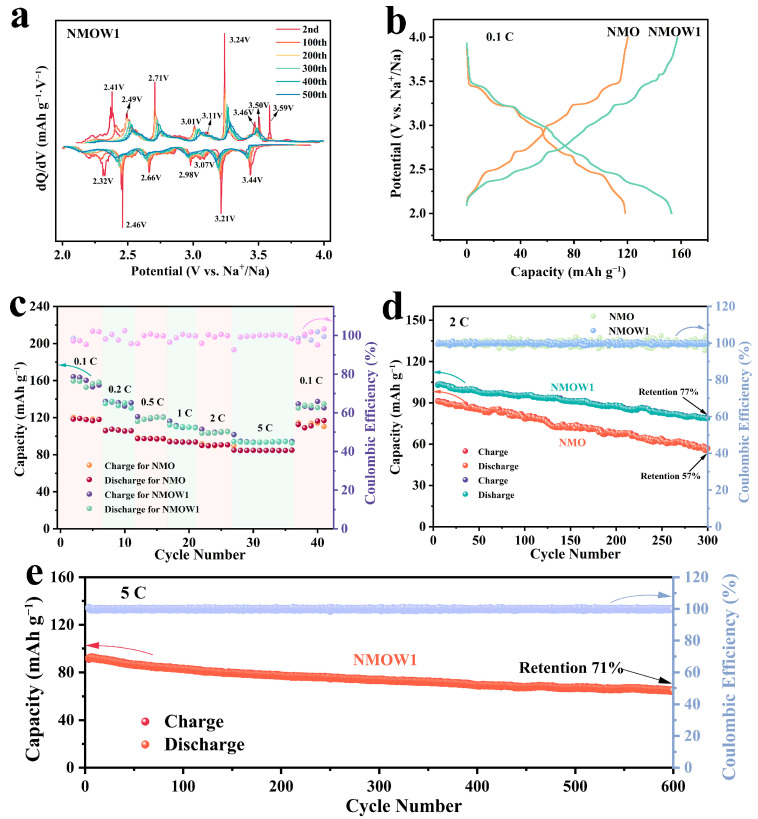
(**a**) dQ/dV curves of NMOW1 between 2.0 and 4.0 V. (**b**) GCD profiles of NMO and NMOW1 at 0.1 C for the second cycle. (**c**) Rate properties of the NMO and NMOW1 from 0.1 to 5 C. (**d**) Cycling property and Coulombic efficiency of NMO and NMOW1 at 2 C. (**e**) Cycle life of NMOW1 at 5 C.

The sodium storage performance of the NMO and NMOW cathodes are summarized in Figure 4 and Figures S4–S7. The dQ/dV profiles of the NMOW1 exhibit various pairs of reversible redox peaks in the range of 2.0 to 4.0 V (Figure 4a), which are in good agreement with the galvanostatic charge/discharge (GCD) profiles that identify the multi-step plateaus (Figure 4b). This phenomenon illustrates that the NMOW1 cathode undergoes a complex multiphase transformation during the sodiation/desodiation processes [14]. Figure S4a shows the cyclic voltammetry (CV) profiles for NMO and NMOW1, where the NMOW1 cathode displays a larger enclosed area in the CV curve than does the NMO example, indicating that the NMOW1 possesses a higher capacity due to the presence of a small proportion of the layered phase. Notably, the redox peak positions and shapes for NMOW1 are almost identical to those of NMO, as the tunnel phase is predominant. According to the redox and phase transition mechanisms, the redox peaks at the high potential range (2.5–4.0 V) are related to the Na^+^/vacancy ordering transition [50,51]. Other peaks in the low potential region (below 2.5 V) are correlated with the Mn^3+^/Mn^4+^ redox reactions [37,38,52]. In addition, the CV profiles of the NMOW1 cathode for the first six cycles overlap well (Figure S4b), manifesting high reversibility and low polarization.

The GCD curves of NMO and NMOW1 at 0.1 C for the second cycle are illustrated in Figure 4b. Both the NMO and NMOW cathodes display similar GCD profiles, with more than six plateau features, which are in accordance with the CV results. The NMOW1 cathode exhibits a higher capacity of 157.3/153.1 mAh g^−1^ compared to that of the NMO (120.1/118.3 mAh g^−1^) example. Meanwhile, the capacities for NMOW0.5, NMOW1.5, and NMOW2 are 129.6/129.3, 127.2/124.1, and 121.5/120.5 mAh g^−1^ for the second cycles at 0.1 C, respectively (Figure S4c). The initial charge/discharge profiles for the as-prepared cathodes at 0.1 C are displayed in Figure S4d. Since the synthesized materials are Na-deficient, the initial charge capacity is lower than the discharge capacity [13]. Moreover, NMOW2 shows a decrease in charge/discharge capacity compared to that of other W-doped samples, which may be attributed to the increase in Na_2_WO_4_ as the W concentration increases [14]. Therefore, the abovementioned results demonstrate that a suitable amount of W-doping could be favorable for enhancing the charge/discharge capacity of the tunnel-type cathode due to the induction of the formation of a small proportion of the layered phase.

The rate performances of the NMO and NMOW cathodes are presented in Figure 4c and Figure S5. Notably, the NMOW1 cathode exhibits outstanding rate performance, with average discharge capacities of 157.2, 134.4, 118.8, 110.2, 103.9, and 93.6 mAh g^−1^ at 0.1, 0.2, 0.5, 1, 2, and 5 C, respectively. The cycling properties of the synthesized samples are tested at 2 C (Figure 4d and Figure S6). The NMOW1 cathode shows a high second charge/discharge capacity of 103.3/102.7 mAh g^−1^ and a superior capacity retention of 77% over 300 cycles compared to the results for the NMO (91.6/91.0 mAh g^−1^, 57% capacity retention), along with a Coulombic efficiency close to 100%. As shown in Figure S6, the NMOW0.5, NMOW1.5, and NMOW2 cathodes exhibit second charge/discharge capacities of 95.2/95.0, 85.9/85.6, and 89.2/88.6 mAh g^−1^ with the corresponding capacity retention of 80%, 71%, and 47% over 300 cycles at 2 C, respectively. Meanwhile, Figure S7 displays the cycling stability for the NMO and NMOW1 cathodes at 1 C, where the NMOW1 cathode obtains a discharge capacity of 72.1 mAh g^−1^ over 300 cycles, significantly outperforming the NMO (62.2 mAh g^−1^) example. Moreover, the NMOW1 cathode also possesses superb long-term cycle stability, with 71% capacity retention over 600 cycles at 5 C (Figure 4e). According to the above results, the layered/tunnel biphasic composite NMOW1 can exhibit higher capacity, better rate performance, and greater cycle stability than pure tunnel phase NMO, originating from the synergistic effects between the layered and tunnel structure, resulting in good potential for practical applications.

**Figure 5:**
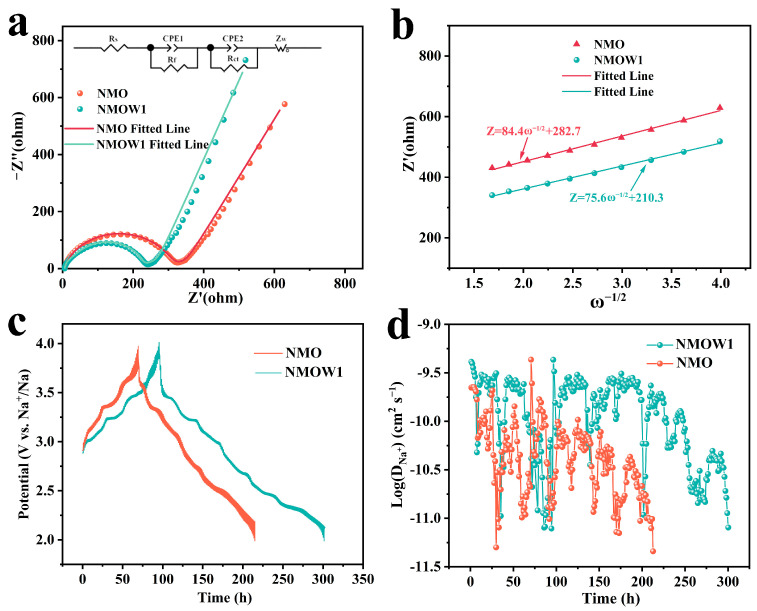
(**a**) Nyquist plots of NMO and NMOW1 after five cycles at 2 C. (**b**) The relationship of Z′ and ω^−1/2^ at low-frequency. GITT profiles (**c**) and the Na^+^ diffusion coefficients (**d**) of NMO and NMOW1.

Electrochemical impedance spectroscopy (EIS) was performed to explore the charge transfer and Na^+^ diffusion kinetics of the NMO and NMOW1 cathodes. All the Nyquist plots consist of one semicircle and a sloping line (Figure 5a). The electrolyte resistance (*R_s_*), electrode/electrolyte interface resistance (*R_e_*), and charge transfer resistance (*R_ct_*) are obtained by the intercept at the real axis Z′ and the semicircle in the high-medium frequency regions [33]. The sloping line at low frequency relates to Warburg impedance (Z*_w_*), correlating with ion migration behavior [14]. The *R_s_*, *R_e_*, and *R_ct_* values of the NMOW1 cathode are 5.4, 22.5, and 190.9 Ω (Table S2), which are much lower than those of NMO (5.5, 48.5, and 240.2 Ω), suggesting an enhanced charge transfer. Furthermore, the NMOW1 exhibits a lower Warburg coefficient (σ) compared to that of NMO (Figure 5b, Table S2), indicating fast Na^+^ transport. Additionally, the galvanostatic intermittent titration technique (GITT) was further employed to evaluate the Na^+^ diffusion coefficient (*D*_Na_^+^). Details of the *D*_Na_^+^ equation can be found in the Supporting Information (Figure S8). The GITT profiles of NMO and NMOW1 are depicted in Figure 5c. The average *D*_Na_^+^ value for NMOW1 (1.5 × 10^−10^ cm^2^ s^−1^) is remarkably higher than that for NMO (5.7 × 10^−11^ cm^2^ s^−1^) (Figure 5d). These results suggest that NMWO1, with the layered/tunnel biphasic structure, possesses superior charge and ion diffusion kinetics, contributing to its outstanding rate performance.

**Figure 6:**
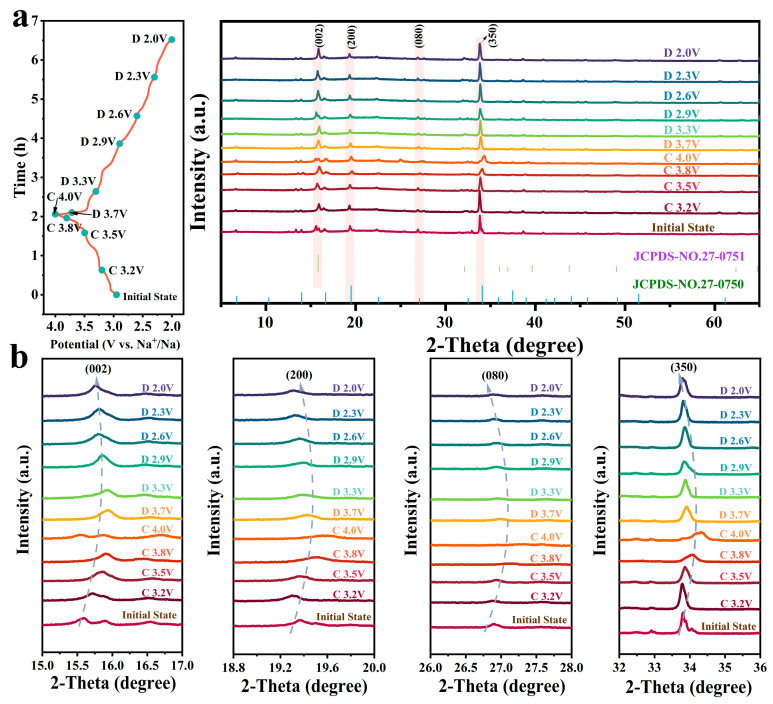
(**a**) Ex situ XRD patterns and corresponding potential plots for NMOW1. (**b**) Enlargement of ex situ XRD patterns region at 15.0−17.0°, 18.8−20.0°, 26.0−28.0°, and 32.0−36.0° for the NMOW1 cathode during the sodiation/desodiation process.

Phase transformation of the NMOW1 material in the Na^+^ insertion/extraction processes was studied using ex situ XRD during the first cycles at different voltage states. As depicted in Figure 6a, the NMOW1 cathode primarily exhibits typical diffraction peaks corresponding to the tunnel and P2-type layered structures. Specifically, the (002) diffraction peak from 15.0° to 17.0° is indexed to the P2 layered structure (Figure 6b). This peak shifts slightly with the potential increasing from 3.2 to 4 V due to more Na^+^ being continuously extracted, accompanying the emergence of the intermediate OP4 phase between the P2 and O2 phases [14,38,52]. Meanwhile, the major characteristic diffraction peaks for (200), (080), and (350) are attributed to the typical tunnel phase. These peaks shift to higher angles during the Na^+^ deintercalation process, indicating a contraction of the lattice parameters in the tunnel structure [25,53]. Subsequently, these peaks gradually return to their original positions during the Na^+^ intercalation process. These results further validate the outstanding structural reversibility and stability of the NMOW1 cathode, contributing to its impressive cycle performance.

**Figure 7:**
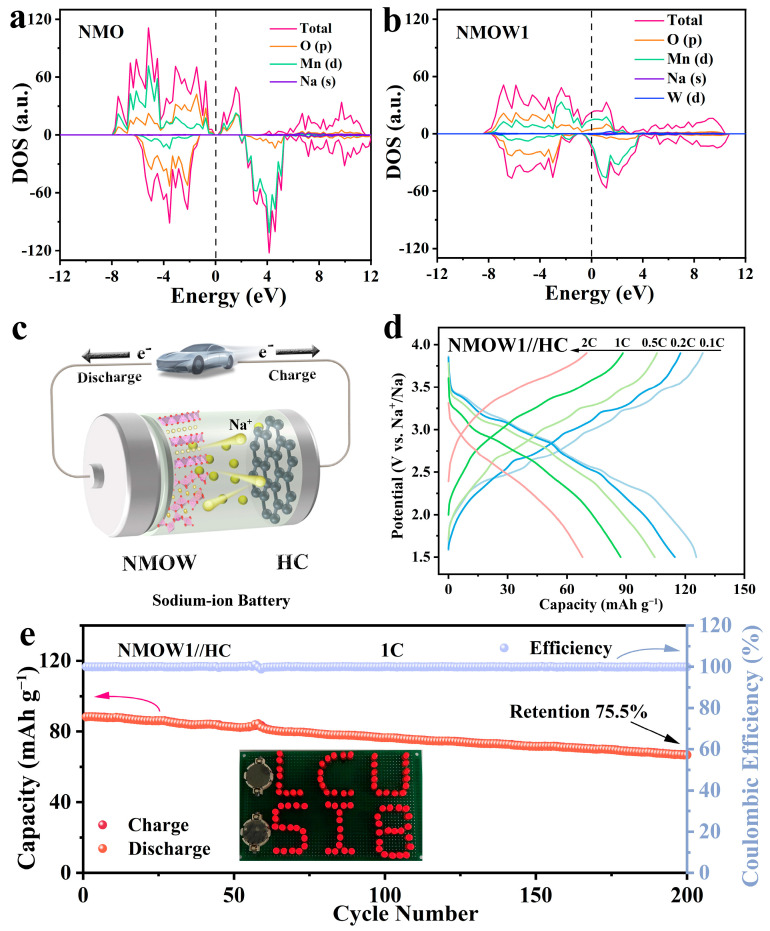
DOS calculation of NMO (**a**) and NMOW1 (**b**). (**c**) Schematic diagram of sodium-ion full cell. (**d**) GCD curves of the NMOW1//HC device at various current rates. (**e**) Cycling capability of the NMOW1//HC device at 1 C, and the illustration shows the LED lights lit up by the NMOW1//HC.

To investigate the effect of W-doping on the electronic structure of the tunnel-type material, DFT calculations were performed on the NMO and NMOW1. The density of states (DOS) for NMO and NMOW1 are depicted in Figure 7a,b. It can be observed that the energy levels near the Fermi level are predominantly dominated by the Mn 3*d* and O 2*p* orbitals for both NMO and NMOW1. Importantly, no band gaps are observed in the DOS for NMOW1, and more electrons gather around the Fermi level compared to the results for NMO, indicating an enhanced electronic conductivity [25,31,54]. This demonstrates that the W-substitution efficiently tunes the electronic structure and redistributes the electronic configuration for the tunnel-type cathode, which results in enhanced Na^+^ (de)intercalation kinetics and excellent rate performance.

To validate the potential application of the NMOW1 sample, a full cell, as depicted in the schematic of Figure 7c, was assembled using the NMOW1 cathode and the commercial hard carbon (HC) anode (denoted as NMOW1//HC). Figure S9a reveals that the NMOW1//HC device delivers an initial capacity of 128.9/125.6 mA h g^−1^ at 0.1 C, with the Coulombic efficiency of 97.4%, based on the cathode active material mass. The rate performance of the NMOW1//HC is illustrated in Figure 7d, delivering reversible capacities of 128.9/125.6, 117.5/114.7, 105.7/104.5, 88.3/87.2, and 69.9/67.9 mAh g^−1^ at 0.1, 0.2, 0.5, 1, and 2 C, respectively. Remarkably, the full cell exhibits splendid long-term cycling stability, maintaining 86.5% and 75.5% capacity retention over 100 and 200 cycles at 1 C, respectively (Figure 7e), which is comparable to or exceeding that of previous reports, as listed in Table S3. Furthermore, the full cell achieves a promising energy density of 183.2 Wh kg^−1^ at 21.1 W kg^−1^, based on the total mass of the cathode and anode active materials. Importantly, as shown in Figure 7e (inset images), the full cell, when charged to 4.0 V, can easily illuminate red-light-emitting diode (LED) bulbs with “LCU” and “SIB” shapes using two independent coin cells, indicating its great potential for practical application.

## 3. Materials and Methods

### Materials Preparation

Following a standard procedure, stoichiometric ratios of CH_3_COONa (AR), Mn(CH_3_COO)_2_·4H_2_O (AR, 99%), and Na_2_WO_4_·2H_2_O (AR) were thoroughly dissolved in oxalic acid solution under continuous stirring for 5 h. All relevant reagents were purchased from Sinopharm Chemical Reagent Co., Ltd (Shanghai, China) and used without any purification. The resulting homogeneous solution was subsequently placed into the oven at 80 °C for 12 h, followed by grinding the agglomerated materials using an agate mortar for 0.5 h. After that, the homogenous precursor underwent calcination in a muffle furnace at 900 °C for 6 h under an air atmosphere. The obtained cathode materials of Na_0.44_Mn_1−x_W_x_O_2_ (x = 0.005, 0.01, 0.015, 0.02) were designated as NMOW0.5, NMOW1, NMOW1.5, and NMOW2, respectively. Additionally, the NMOW1 cathode with other annealed temperatures (700, 800, and 1000 °C) and times (3, 9, 12, and 15 h) was synthesized using identical routes. The undoped Na_0.44_MnO_2_ (NMO) material was prepared via the analogous method, as described in our previous work [27].

Additional detailed information about material characterization, electrochemical measurements, and DFT calculation is provided in the Supporting Information.

## 4. Conclusions

In summary, NMOW cathodes with layered/tunnel biphasic structures were successfully prepared through a realizable co-precipitation method by controlling the tungsten-doping ratio, annealing temperature, and time. As a result, a tunnel-dominated composite cathode can be successfully fabricated using this synthetic route. Moreover, the introduction of W not only significantly increases the electron density around the Fermi level and improves the metallicity of the tunnel-type material but also efficiently promotes the Na^+^ diffusion kinetics and maintains highly reversible structural conversion during repetitive sodiation/desodiation processes. Accordingly, the optimized biphasic structures of the NMOW1 cathode integrate the superiorities of the high capacity of the layered structure and the superb cycle performance of the tunnel structure, delivering a high reversible capacity, superior rate performance, and outstanding cycling life. More importantly, the NMOW1//HC device displays a promising energy density of 183.2 Wh kg^−1^ at 21.1 W kg^−1^, accompanied by excellent capacity retention. This study offers a guiding significance for the development of hybrid structure cathodes, mainly composed of the tunnel phase, through trace cation substitution and a reasonable preparation strategy, propelling the practical application of high-performance SIBs.

## Data Availability

Data will be made available on request.

## Supplementary Materials

The following supporting information can be downloaded at: https://www.mdpi.com/article/10.3390/molecules30102175/s1, Figure S1: The crystal structure of the tunnel phase (a) and the layered phase (b). XRD images of the NMOW1 cathode under different annealing times (c) and temperatures (d); Figure S2: SEM images of NMO, NMOW0.5, NMOW1.5, and NMOW2; Figure S3: SEM images of NMOW1 3 h, NMOW1 9 h, NMOW1 12 h, NMOW1 15 h, NMOW1 700 °C, NMOW1 800 °C, and NMOW1 1000 °C; Figure S4: (a) CV curves of NMO and NMOW1 at 0.2 mV s^−1^ for the second cycle. (b) CV curves of NMOW1 at 0.1 mV s^−1^ for the first six cycles. (c) GCD curves of the NMOW0.5, NMOW1.5, and NMOW2 at 0.1 C for the second cycle. (d) GCD profiles of NMO, NMOW0.5, NMOW1, NMOW1.5, and NMOW2 at 0.1 C for the first cycle; Figure S5: Rate performance of NMOW0.5 (a), NMOW1.5 (b), and NMOW2 (c); Figure S6: Cycling stability and Coulombic efficiency of NMOW0.5 (a), NMOW1.5 (b), and NMOW2 (c) at 2 C; Figure S7: Cycling stability and Coulombic efficiency of NMO and NMOW1 at 1 C; Figure S8: A single-step titration of NMO (a) and NMOW1 (c) during GITT measurement. The linear relationship of NMO (b) and NMOW1 (d) between the potential and τ^1/2^ during the titration; Figure S9: (a) Initial charge/discharge profile of the NMOW1 cathode and the NMOW1//HC full cell at 0.1 C. (b) Initial charge/discharge profile of the HC anode at 30 mA g^−1^; Table S1: Summary of fitted parameters in Rietveld refinement of XRD pattern; Table S2: The fitting results of the equivalent circuit from the EIS curves; Table S3: Comparison of electrochemical performance between NMOW1//HC and the previously reported SIBs full cell. Refs [55,56,57,58,59,60,61,62,63,64,65,66] are cited in the Supplementary Materials.
