# Supplementary material for: Constructing Layered/Tunnel Biphasic Structure via Trace W-Substitution in Tunnel-Type Cathode for Elevating Sodium Ion Storage
Source: Molecules. 2025 May 15;30(10):2175. doi: 10.3390/molecules30102175 (PMC12113807; doi:10.3390/molecules30102175)
Supplement: Supplementary file 1 [file molecules-30-02175-s001.zip › molecules-3596836-supplementary.pdf]

Supporting Information

Constructing Layered/Tunnel Biphasic Structure via Trace W-Substitution in Tunnel-Type Cathode for Elevating Sodium Ion Storage

Wenjing Shi [a,*], Hengxiang Li [a,*], Zihan Wang [a], Lingyang Liu [a], Yixin Feng [a], Rui Qiao [a], Ding Zhang [b], Haibo Li [a], Zhaoyang Wang [a], Pengfang Zhang [a]

[a] *Shandong Provincial Key Laboratory of Chemical Energy Storage and Novel Cell Technology, School of Chemistry and Chemical Engineering, Liaocheng University, Liaocheng 252059, China*

[b] *School of Chemical Engineering and Pharmacy, Wuhan Institute of Technology, Wuhan 430205, China*

* Correspondence: swjuser@163.com (W.S.); lihengxiangg@163.com (H.L.)

1. Material characterization

The character structural information of the as-prepared samples was examined using X-ray diffraction (XRD) analysis with Cu Kα radiation (λ=0.15406 nm) on a Rigaku SmartLab 9 kW instrument (Tokyo, Japan). The morphologies and elemental composition were observed by scanning electron microscopy (SEM, GX4, Waltham, MA, USA) and transmission electron microscopy (TEM, F200X, Talos, Waltham, MA, USA) equipped with energy dispersive spectroscopy (EDS). The chemical composition was characterized by X-ray photoelectron spectroscopy (XPS, Escalab Xi+, Waltham, MA, USA).

2. Electrochemical measurements

The cathode materials (80 wt%), Super P (10 wt%), and polyvinylidene fluoride (PVDF, 10 wt%) were mixed and ground by adding N-methyl-pyrrolidinone (NMP) to form a homogeneous slurry. Then, the obtained slurry was coated on the Al foil and dried at 100 °C for 12h in a vacuum oven. The electrode was cut into a circular disk with 12 mm. The half-cell was assembled with CR2032 coin cells in an Ar-filled glove box (H_2O, O_2<0.01 ppm) by using NMOW or NMO as working electrode, 1.0 M $NaClO_4$ in ethylene carbonate and propylene carbonate (1:1 in volume) with 2 wt% fluoroethylene carbonate as the electrolyte, sodium metal as the counter electrode, and glass fiber (Whatman) as the separator, respectively. In addition, the full-cell was assembled with the same as the half-cell process expect for the anode used the commercial hard carbon. The commercial hard carbon (80 wt%), Super P (10 wt%), and PVDF (10 wt%) were ground by adding the NMP solution to form the slurry. Then,

the obtained slurry was coated on the Cu foil and dried at 100 °C for 12 h in a vacuum oven. The electrode was also cut into 12 mm circular disk. Before assembling NMOW1//HC full-cell, the NMOW cathode and hard carbon anode were pre-cycled for 3 cycles at 0.1 C and 30 mA g^{-1}, respectively. The capacity ratio of the negative to positive electrodes is adjusted to about 1.2. The cyclic voltammetry (CV) between 2 to 4 V for half-cell, and electrochemical impedance spectroscopy (EIS) with an amplitude of 5 mV from 0.01 Hz to 100 kHz were performed by CHI1020C and CHI660E (Chenhua, Shanghai) electrochemical workstation. Galvanostatic charge-discharge (GCD) was carried out on the LAND CT3002A testing system in the voltage range of 2.0–4.0 V (*vs.* Na^+/Na). Galvanostatic intermittent titration technique (GITT) measurements were performed with a charging/discharging at 0.1 C for 4 min and a relaxation time for 60 min.

3. The density functional theory (DFT) calculation

The present DFT calculations are performed by the Vienna *ab initio* Simulation Package (VASP) [1] with the projector augmented wave (PAW) method [2]. The exchange functional is treated using the generalized gradient approximation (GGA) of Perdew-Burke-Emzerhof (PBE) [3] functional. The energy cutoff for the plane wave basis expansion was set to 450 eV and the force on each atom less than 0.05 eV $Å^{-1}$ was set for the convergence criterion of geometry relaxation. Grimme's DFT-D3 methodology [4] was used to describe the dispersion interactions. Partial occupancies of the Kohn−Sham orbitals were allowed using the Gaussian smearing method and a width of 0.05 eV. The Brillouin zone was sampled with a Monkhorst mesh 3×3×1

through all the computational processes. The self-consistent calculations apply a convergence energy threshold of 10^{-5} eV.

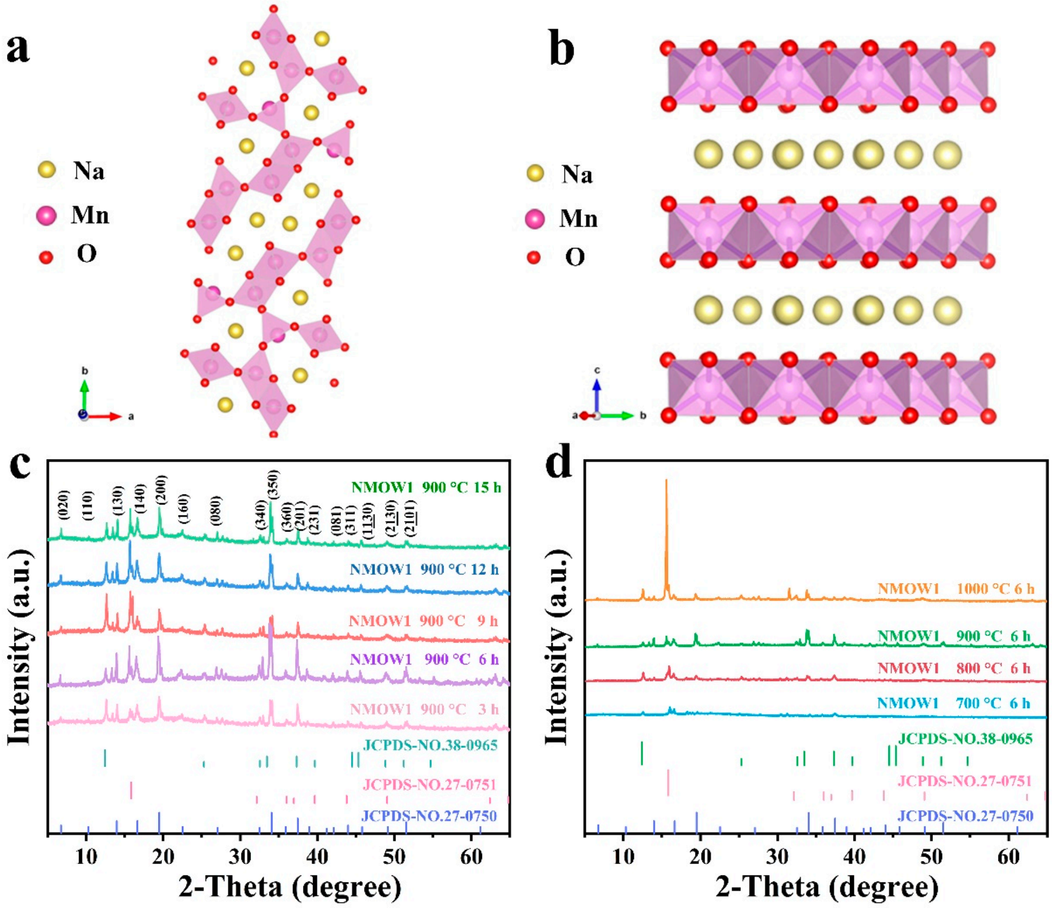

Figure S1. The crystal structure of the tunnel phase (a) and the layered phase (b). XRD images of the NMOW1 cathode under different annealing times (c) and temperatures (d).

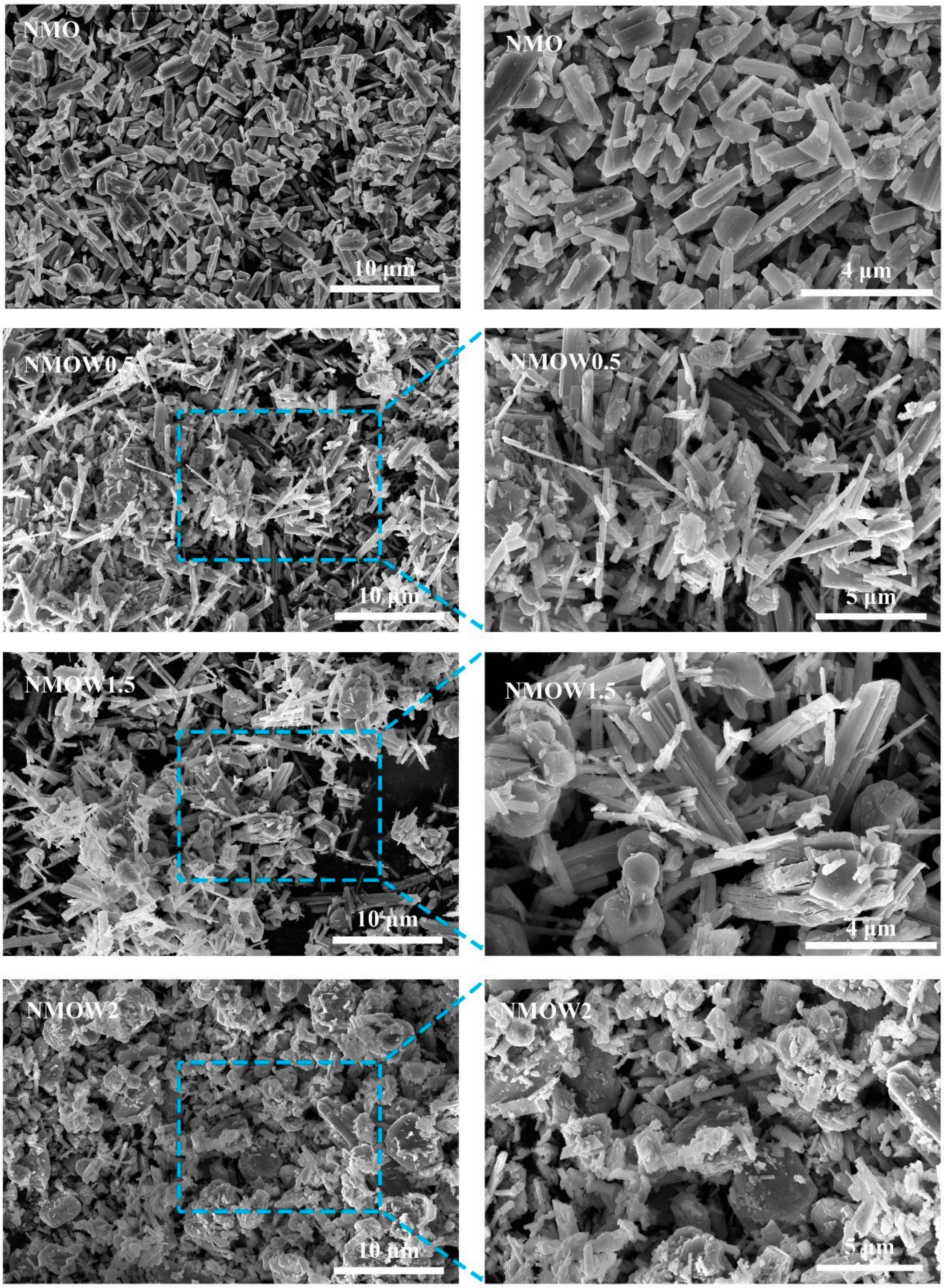

Figure S2. SEM images of NMO, NMOW0.5, NMOW1.5, and NMOW2.

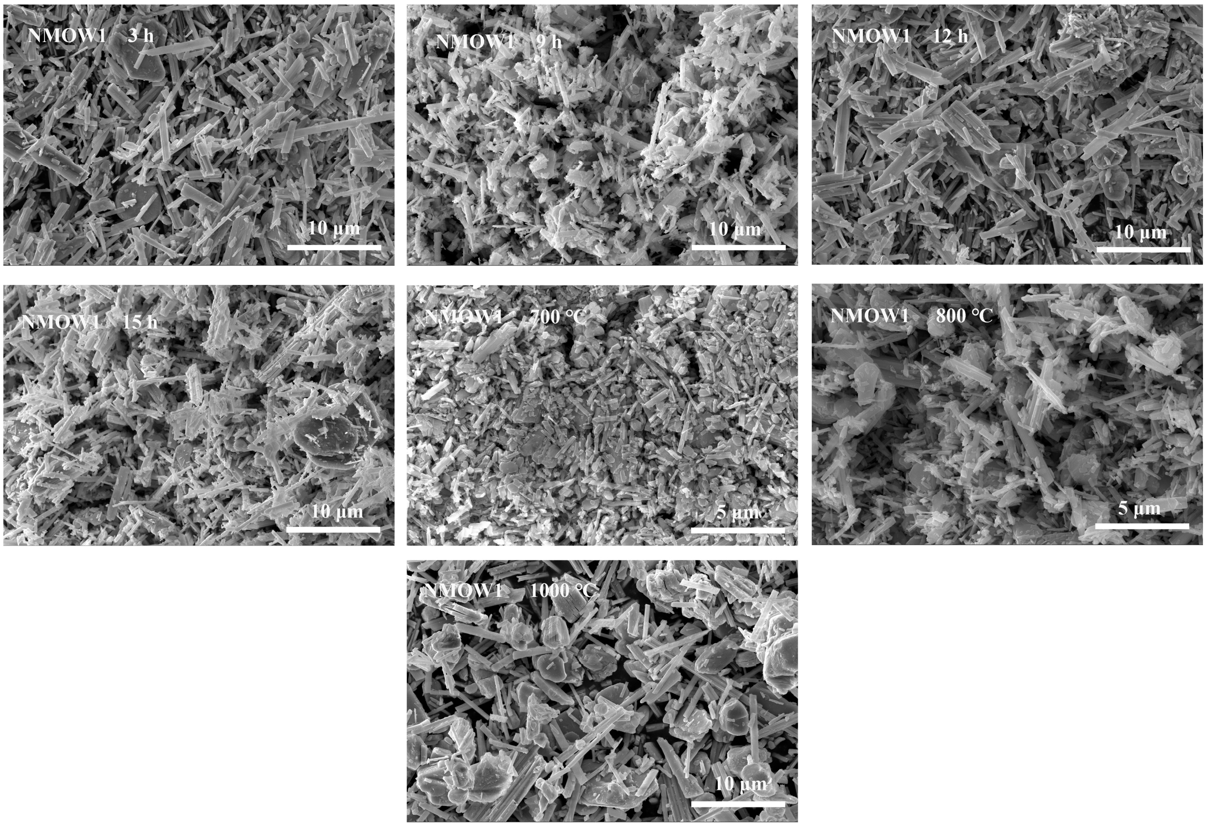

Figure S3. SEM images of NMOW1 3 h, NMOW1 9 h, NMOW1 12 h, NMOW1 15 h, NMOW1 700 °C, NMOW1 800 °C, and NMOW1 1000 °C.

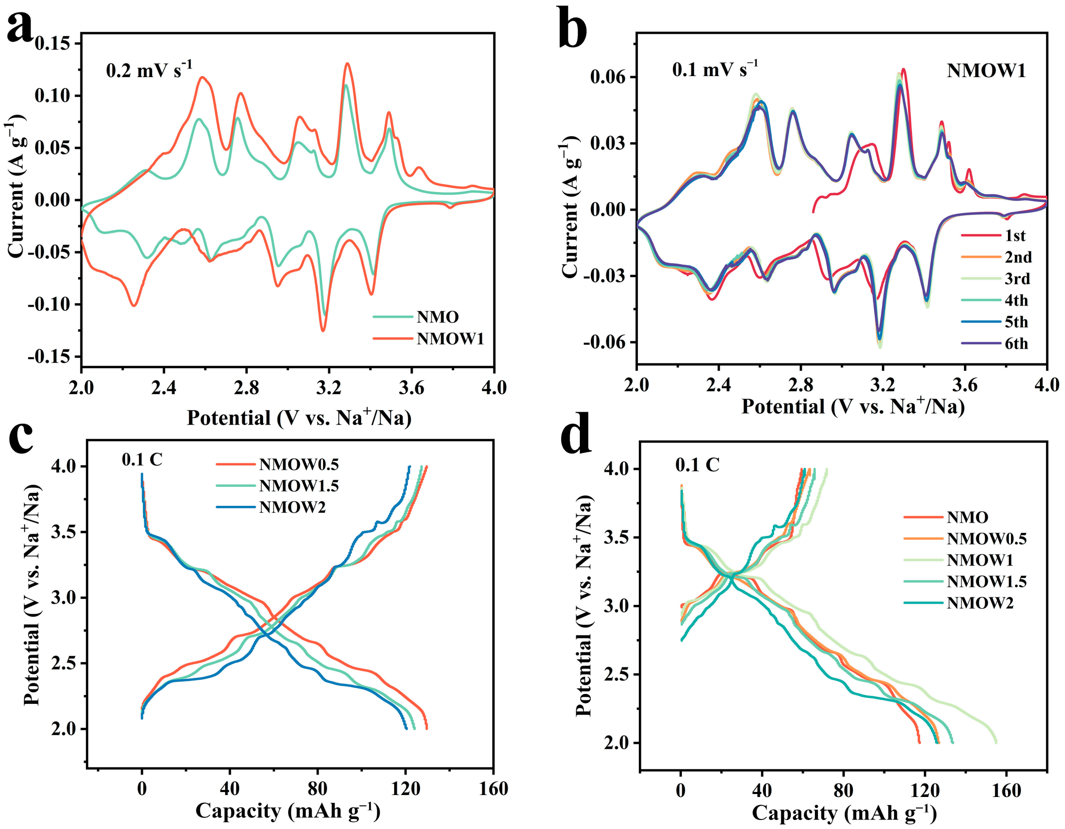

Figure S4. (a) CV curves of NMO and NMOW1 at 0.2 mV s^{-1} for the second cycle. (b) CV curves of NMOW1 at 0.1 mV s^{-1} for the first six cycles. (c) GCD curves of the NMOW0.5, NMOW1.5, and NMOW2 at 0.1 C for the second cycle. (d) GCD profiles of NMO, NMOW0.5, NMOW1, NMOW1.5, and NMOW2 at 0.1 C for the first cycle.

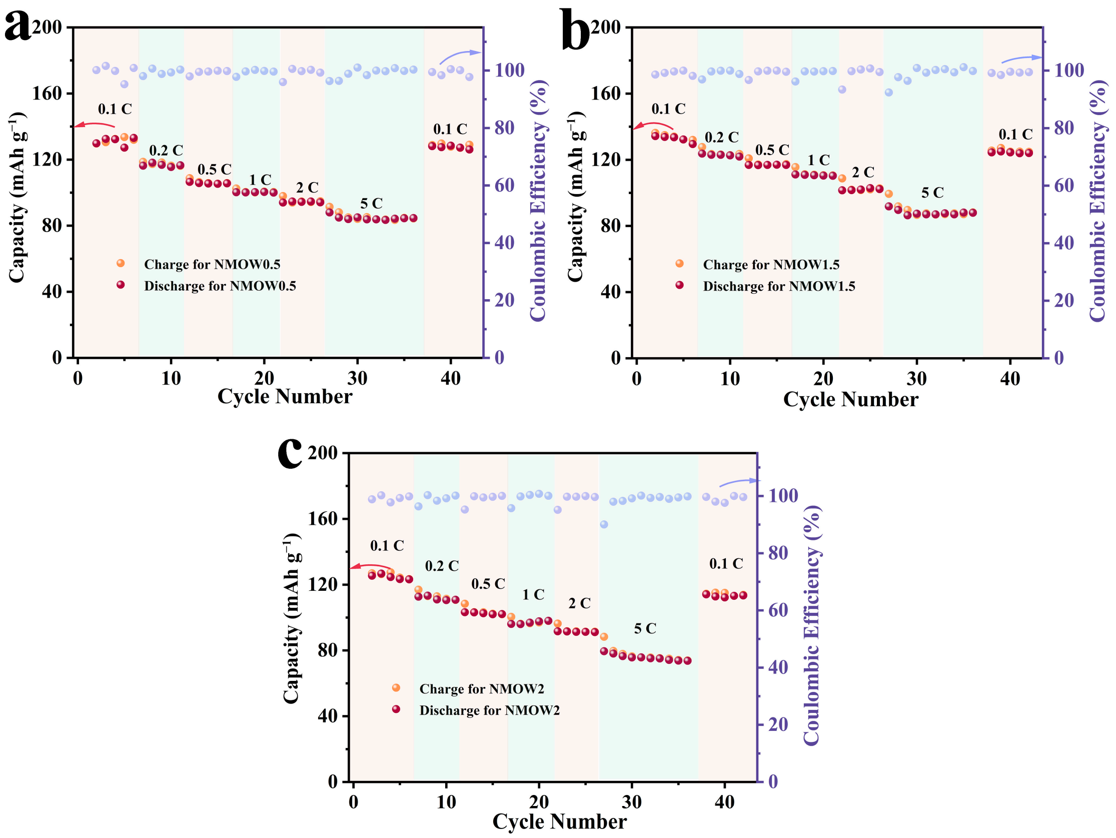

Figure S5. Rate performance of NMOW0.5 (a), NMOW1.5 (b), and NMOW2 (c).

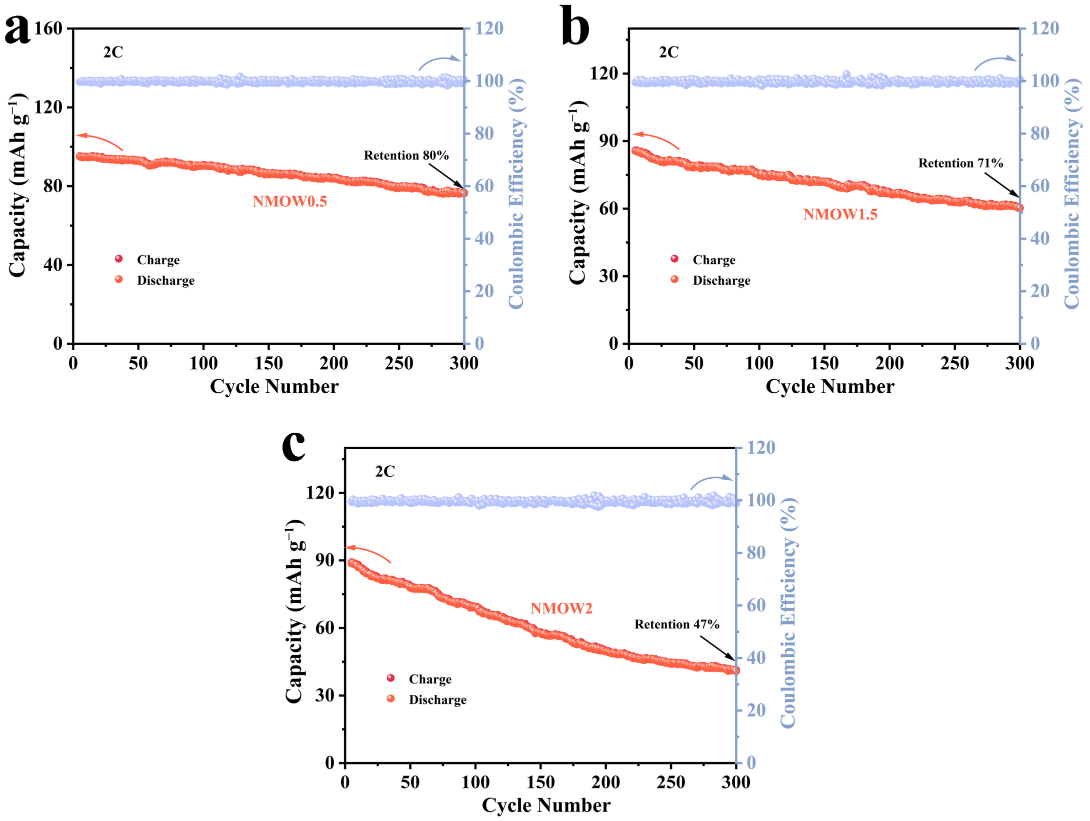

Figure S6. Cycling stability and Coulombic efficiency of NMOW0.5 (a), NMOW1.5 (b), and NMOW2 (c) at 2 C.

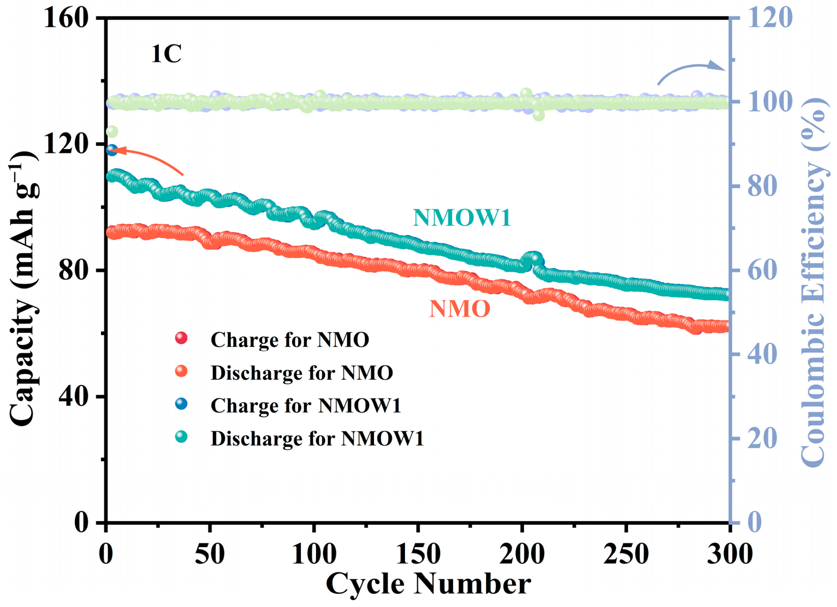

Figure S7. Cycling stability and Coulombic efficiency of NMO and NMOW1 at 1 C.

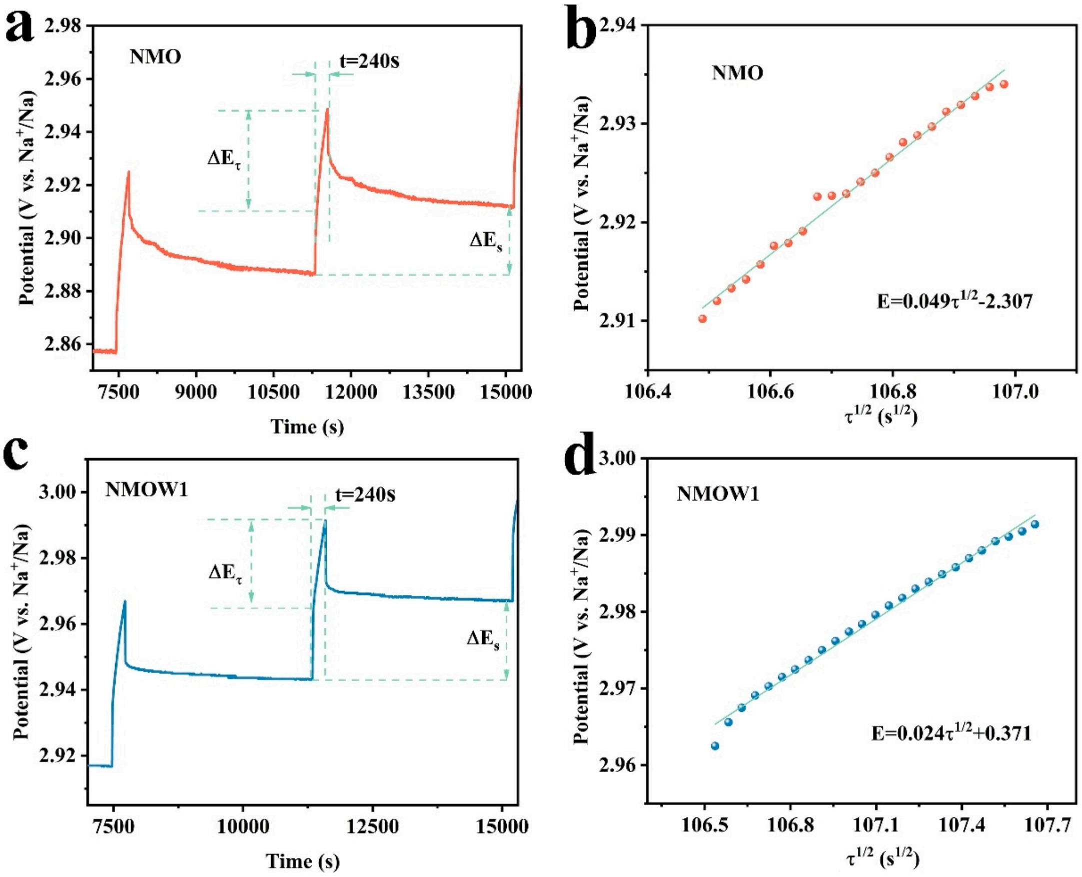

Figure S8. A single-step titration of NMO (a) and NMOW1 (c) during GITT measurement. The linear relationship of NMO (b) and NMOW1 (d) between the potential and $\tau^{1/2}$ during the titration.

The diffusion behaviors of the NMO and NMOW1 cathodes were evaluated using the GITT. The cells were charged/discharged for 4 min and then relaxed for 60 min to make the voltage reach equilibrium (Figure S8a, c). The Na^+ diffusion coefficient (D_{Na^+}) is assumed to meet Fick's second law of diffusion. When the relationship between potential and $\tau^{1/2}$ shows a linear behavior (Figure S8b, d), the D_{Na^+} in the single-phase regions can be calculated by Equation (S1):

$$D_{Na^+} = \frac{4}{\pi\tau}\left(\frac{m_B V_M}{M_B S}\right)^2 \left(\frac{\Delta E_S}{\Delta E_\tau}\right)^2 \quad \text{(S1)}$$

where m_B, V_M, and M_B are the mass, molar volume, and molecular weight of the active materials. S is the geometric area of the electrode. ΔE_S is the change of the steady-state voltage, and ΔE_τ is the total voltage change during the constant current pulse time.

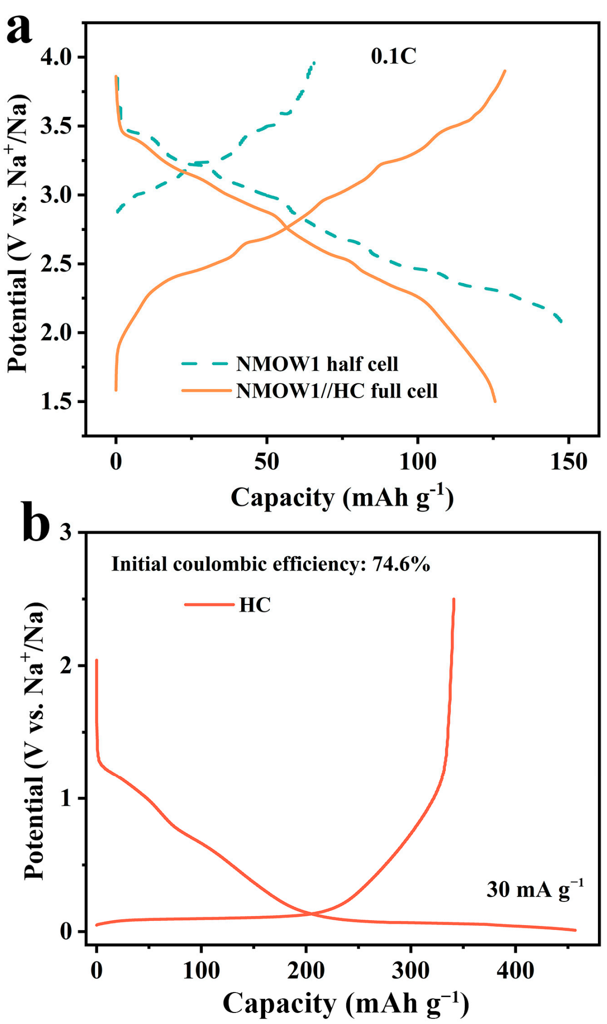

Figure S9. (a) Initial charge/discharge profile of the NMOW1 cathode and the NMOW1//HC full cell at 0.1 C. (b) Initial charge/discharge profile of the HC anode at 30 mA g^{-1}.

Table S1. Summary of fitted parameters in Rietveld refinement of XRD pattern.

Composition	*a* (Å)	*b*(Å)	*c*(Å)	V(Å^3)
NMO[a]	9.0810(3)	26.4513(4)	2.8229(9)	678.0726
NMOW1	9.0812(5)	26.4482(9)	2.8232(2)	678.0833

a: Rietveld refinement results of NMO were obtained from our previous work [5].

Table S2. The fitting results of the equivalent circuit from the EIS curves

Electrode	R_s(Ω)	R_e(Ω)	R_{ct}(Ω)	σ
NMO	5.5	48.5	240.2	84.4
NMOW1	5.4	22.5	190.9	75.6

Table S3. Comparison of electrochemical performance between NMOW1//HC and the previously reported SIBs full cell.

Full cell	Current density	Cycle number	Capacity retention	Energy density (Wh kg^{-1})	Power density (W kg^{-1})	Ref.
MT10//HC	0.2 C	100 cycles	75.7%	-	-	[6]
LT-NaMO//HC	1 C	100 cycles	85.0%	3 C 184.5	-	[7]
O3-$Na[Li_{0.05}Mn_{0.50}Ni_{0.30}Cu_{0.10}Mg_{0.05}]O_2$//HC	0.5 C	100 cycles	~50%	215	-	[8]
LT-NFM//HC	1 C	100 cycles	81.9%	186.2	60.3	[9]
P2-$Na_{2/3}Ni_{1/3}Mn_{2/3}O_2$//HC	0.1 C	50 cycles	85%	212.5		[10]
O3-NFMCu-0.2//HC	0.2 C	100 cycles	~47.1%	-	-	[11]
P2-NNZMTOF//HC	1 C	100 cycles	66.1%		-	[12]
NMM-0.05//HC	0.2 C	50 cycles	77.5%	-	-	[13]
O3-NNAMO//HC	0.5 C	200 cycles	66.5%	213.5	49.1	[14]
**NMOW1//HC**	**1 C**	**100 cycles**	**86.5%**	**0.1 C 183.2**	**21.1**	**This work**
		**200 cycles**	**75.5%**	**1 C 153.4**	**207.7**	

References

[1] Kresse, G.; Furthmiiller, J. Efficiency of ab-initio total energy calculations for metals and semiconductors using a plane-wave basis set. *Comp. Mater. Sci.* **1996**, 6 15–50.

[2] Blochl, P.E. Projector augmented-wave method, *Phys. Rev. B* **1994**, 50, 17953–17979.

[3] Perdew, J.P.; Chevary, J.A.; Vosko, S.H.; Jackson, K.A.; Pederson, M.R.; Singh, D.J.; Fiolhais, C. Atoms, molecules, solids, and surfaces: applications of the generalized gradient approximation for exchange and correlation, *Phys. Rev. B* **1992**, 46, 6671–6687.

[4] Grimme, S.; Antony, J.; Ehrlich, S.; Krieg, H. A consistent and accurate ab initio parametrization of density functional dispersion correction (DFT-D) for the 94 elements H-Pu, *J. Chem. Phys.* **2010**, 132 154104.

[5] Shi, W.J.; Li, H.X.; Zhang, D.; Du, F.H.; Zhang, Y.H.; Wang, Z.Y.; Zhang, X.H.; Zhang, P.F. Insights into unrevealing the effects of the monovalent cation substituted tunnel-type cathode for high-performance sodium-ion batteries, *Chem. Eng. J.* **2023**, 477, 146976.

[6] Wang, D.; Shi, C.; Deng, Y.P.; Wu, Z.; Yang, Z.; Zhong, Y.; Jiang, Y.; Zhong, B.; Huang, L.; Guo, X.; Chen, Z. A fundamental understanding of the Fe/Ti doping induced structure formation process to realize controlled synthesis of layer-tunnel $Na_{0.6}MnO_2$ cathode, *Nano Energy* **2020,** 70, 104539.

[7] Su, Y.; Zhang, N.N., Li, J.Y.; Liu, Y.; Hu, H.Y.; Wang, J.; Li, H.; Kong, L.Y.; Jia, X.B.; Zhu, Y.F.; Chen, S.; Wang, J.Z.; Dou, S.X.; Chou, S.; Xiao, Y. Sodium layered/tunnel intergrowth oxide cathodes: Formation process, interlocking chemistry, and electrochemical performance, *ACS Appl. Mater. Interfaces* **2023**, 15, 44839–44847

[8] Deng, J.; Luo, W.-B.; Lu, X.; Yao, Q.; Wang, Z.; Liu, H.-K.; Zhou, H.; Dou, S.-X. High energy density sodium-ion battery with industrially feasible and air-stable O3-type layered oxide cathode, *Adv. Energy Mater.* **2018**, 8, 1701610.

[9] Sun, Z.; Peng, B.; Zhao, L.; Li, J.; Shi, L.; Zhang, G. Constructing layer/tunnel biphasic $Na_{0.6}Fe_{0.04}Mn_{0.96}O_2$ enables simultaneous kinetics enhancement and phase transition suppression for high power/energy density sodium-ion full cell, *Energy Storage Mater.* **2021**, 29, 320–328.

[10] Liu, Y.; Shen, Q.; Zhao, X.; Zhang, J.; Liu, X.; Wang, T.; Zhang, N.; Jiao, L.; Chen, J.; Fan, L.Z. Hierarchical engineering of porous P2-$Na_{2/3}Ni_{1/3}Mn_{2/3}O_2$ nanofibers assembled by nanoparticles enables superior sodium-ion storage cathodes, *Adv. Funct. Mater.* **2020**, 30, 1907837.

[11] Zhang, Z., Liu, Y.; Liu, Z.; Li, H.; Huang, Y.; Liu, W.; Ruan, D.; Cai, X.; Yu, X. Dual-strategy of Cu-doping and O3 biphasic structure enables Fe/Mn-based layered oxide for high-performance sodium-ion batteries cathode, *J. Power Sources* **2023**, 567, 232930.

[12] Fan, Y.; Ye, X.; Yang, X.; Guan, L.; Chen, C.; Wang, H.; Ding, X. Zn/Ti/F synergetic-doped $Na_{0.67}Ni_{0.33}Mn_{0.67}O_2$ for sodium-ion batteries with high energy density, *J. Mater. Chemistry A* **2023**, 11, 3608–3615.

[13] Li, X.L.; Bao, J.; Li, Y.F.; Chen, D.; Ma, C.; Qiu, Q.Q.; Yue, X.Y.; Wang, Q.C.; Zhou, Y.N. Boosting reversibility of Mn-based tunnel-structured cathode materials for sodium-ion batteries by magnesium substitution, *Adv. Sci.* **2021**, 8, 2004448.

[14] Peng, B.; Chen, Y.; Zhao, L.; Zeng, S.; Wan, G.; Wang, F.; Zhang, X.; Wang, W.; Zhang, G. Regulating the local chemical environment in layered O3-$NaNi_{0.5}Mn_{0.5}O_2$ achieves practicable cathode for sodium-ion batteries, *Energy Storage Mater.* **2023**, 56, 631–641.
